# The Effect of Changing the Angle of the Passenger Car Seat Backrest on the Head Trajectories of the 50th Percentile Male Dummy

**DOI:** 10.3390/s24123868

**Published:** 2024-06-14

**Authors:** Damian Frej

**Affiliations:** Department of Automotive Engineering and Transport, Kielce University of Technology, Avenue Tysiaclecia Państwa Polskiego 7, 25-314 Kielce, Poland; dfrej@tu.kielce.pl

**Keywords:** crash tests, car seat, safety, seat belts, anthropometric dummy

## Abstract

The aim of the study is to compare the head displacement of the KPSIT C50 dummy during a frontal collision at a speed of 20 km/h, along with the change in the angle of the car seat backrest. Passenger car manufacturers recommend setting the backrest angle of the car seat between 100 and 125 degrees. It should be noted that the driver’s position is of great importance in the event of a collision injury. In the event of a rear-end collision, the position of the headrest of the car seat is an element that affects the degree of the driver’s injuries. In extreme cases, incorrect positioning of the headrest, even at low speed, can lead to serious injuries to the cervical spine and even death. The article is part of a large-scale study on low-speed crash testing. The research problem concerned the influence of the seat backrest angle on the head displacement during a low-speed collision. The article compares the displacement of the head of the KPSIT C50 dummy during a series of crash tests, where the angle of the car seat backrest was changed. On the basis of the research, it was found that the optimal angle of the car seat backrest is 110 degrees. In addition, a preliminary analysis of the displacements of the dummy’s head showed a high risk of whiplash injury in people sitting in a fully reclined seat.

## 1. Introduction

Road accidents in Poland between 2010 and 2023 constituted a significant problem, similar to many other countries in the European Union. Despite the implementation of various remedial measures and campaigns aimed at improving road safety, the number of accidents remained high [1,2,3]. Together with other EU countries, Poland endeavored to take actions to improve road safety, including educational campaigns, speed controls, initiatives for safe driving practices, and infrastructure enhancements. However, the effectiveness of these actions varied, and the situation in Poland may have been worse or better than the EU average during certain periods. Road accidents not only contribute to human tragedies but also generate significant social and economic costs. Therefore, improving road safety remains a priority for Poland and other EU countries [4,5,6].

Road accidents entail diverse injuries that can significantly impact the lives of victims. Among the most common injuries are head injuries, such as concussions, whiplash, or severe skull damage, which can lead to permanent neurological damage or death. Additionally, severe collisions can cause spinal injuries, such as vertebral fractures, which in turn can result in paralysis or the permanent impairment of motor functions. Limb injuries, including fractures, sprains, or muscle damage, are also common and require immediate medical attention [7,8,9]. Road accidents can also lead to chest injuries, such as rib fractures or lung injuries, significantly impeding breathing and potentially having serious health consequences. There is also a risk of internal injuries, which may be difficult to diagnose at the scene of the accident but can be life-threatening if not properly identified and treated [10,11,12].

It should be noted that the analysis and interpretation of these injuries help to understand the scale of the tragedy and the necessity of taking actions to improve road safety. To accurately understand the injuries accompanying road accidents, numerous simulations of crash tests and physical crash tests with anthropometric dummies must be conducted to identify, understand, and interpret the risks of bodily harm exactly [13,14,15]. Furthermore, implementing effective strategies such as driver education, improving road infrastructure, and promoting innovative safety technologies can help reduce the number of road accidents and mitigate the consequences of injuries for the victims [16,17,18].

Whiplash Associated Disorder (WAD), commonly referred to as neck injury, is a global issue. These injuries occur with relatively minor changes in speed (usually <25 km/h) in impacts from all directions. However, rear-end impacts are most common in injury statistics. Women are 1.5–3 times more prone to neck injuries than men [19,20,21]. It should be noted that in most modern cars, improved seat design is the dominant measure to enhance passenger protection against neck injuries in rear-end collisions. Currently, the exact mechanisms of injury causing the so-called “whiplash injury” are not yet known. Various researchers have proposed a number of hypotheses regarding the nature and location of the injury. Therefore, a number of injury criteria have been assessed to enable researchers to predict injuries in the event of low-speed rear-end collisions [22,23,24].

Active headrests effectively protect the cervical spine from injuries, so they are increasingly being used in modern cars. Depending on the design principle and the mechanism of action, they differ in activation method. For a long time, they were only used in higher-class car seats, but due to their high effectiveness, they are increasingly found in mid-range vehicles. These solutions are recommended by road safety organizations and crash test agencies, such as EuroNCAP, NHTSA, or IIHS. Active headrests are activated during a rear collision. Their operation is automatic and involves a “following” of the passenger’s head as quickly as possible to relieve the cervical spine before potential damage occurs, which increases the effectiveness of the protection. Engineers from SAAB, who designed the popular SAHR system in 1997, are considered pioneers in this field. Today, these solutions appear under various names and, depending on the manufacturer, they differ in activation method. Modern materials, such as expanded polypropylene (EPP), are used in the production of headrests, further improving safety parameters [25,26,27].

While the primary function of active front seat headrests is to adjust to the movement of the passenger’s and driver’s head during a collision, they also require adjustment. In most cases, their height can be adjusted, as well as the tilt—either manually or with the push of a button. It is important to remember that a headrest set too low may not protect against a sudden and forceful backward bending of the head, which can result in serious injuries. Adjusting the driver’s seat and maintaining the correct position behind the wheel are safety prerequisites regardless of whether the car is equipped with an active or passive headrest. Headrests that react in the event of a collision can be categorized as reactive or active. The primary difference between them is the type of activation system. The former operate on a mechanical basis, based on a lever and spring system within the headrest. In contrast, active systems are powered by actuators controlled by an electronic system. The latter are considered faster and more precise [28,29,30].

Unlike active headrests, which react to collision situations and adjust to the passenger’s body movement, passive headrests merely serve the function of statically supporting the head and act as a barrier to prevent it from tilting backward. They can be an extension of the seat or a separate element, similar to an active headrest. However, unlike active headrests, they are generally rigidly attached to the backrest frame and do not have adjustment mechanisms forwards and backward or at an angle. They can only be adjusted for the passenger’s height by raising or lowering them. From the outside, it can sometimes be difficult to distinguish between passive and active headrests, as they may look the same. A visual distinguishing feature of active headrests from passive ones in some car models is their characteristic two-piece construction [31,32,33].

Electric seat adjustment, memory settings, or ventilated and heated car seats are just a few examples of the most desired additional equipment elements in a car. Proper adjustment of the driver’s seat allows for the correct position behind the wheel, which in turn affects driving comfort and safety. Therefore, modern seats are equipped with additional sensors and systems. As much as 52% of new car drivers in 2022 chose this type of additional equipment. It should be noted that if a car is not used by many drivers, such a function will only be useful once. However, there may potentially be more passengers. In such situations, electric seat adjustment will save time on manually readjusting the headrest, backrest, and seat [34,35,36].

The main research objective was to compare changes in the displacement of the KPSIT C50 dummy’s head during a frontal collision at a speed of 20 km/h. For this purpose, the angle of the car seat backrest was adjusted from 100 to 125 degrees in each series of crash tests involving the dummy. This study is part of a comprehensive examination of human body behavior during low-speed crash tests. The experiment aimed to determine the impact of the car seat backrest angle on the trajectory of the dummy’s head movement. Additionally, the experiment sought to identify the most optimal backrest angle for a passenger car seat. The authors aimed to investigate whether a backrest angle above 115 degrees would result in head rotation, as observed in high-speed crash tests described in the literature above 30 km/h.

## 2. Research Background

Nowadays, road safety and the effectiveness of protective equipment in vehicles are key issues. The subject of road accidents and crash tests occupies an important place in vehicle safety research. In this chapter, we will focus on the broad context of these issues, analysing relevant aspects and conclusions from the scientific literature.

The literature review will allow us to explore the following issues: the causes of road accidents, as identifying the main factors leading to road accidents is crucial for the development of effective strategies to prevent them. Crash tests: As a tool for assessing the safety of vehicles, crash tests are an integral part of the process of designing and evaluating vehicles in terms of safety. We will examine different testing methods and how they contribute to improving vehicle safety. Technologies that improve road safety: The development of technologies in vehicles such as emergency braking systems, lane assistants and blind spot monitoring systems have a significant impact on the reduction in road accidents. We will follow the latest developments in this field and their effectiveness. Legal and regulatory aspects: In addition to technological solutions, relevant regulations and regulations also play an important role in improving road safety. We will analyse the existing regulations and possible directions for their further development. By analysing these issues, we will be able to better understand today’s road safety challenges and how to effectively address them.

In article [37], the authors conducted a study aimed at identifying the most common and consistently occurring causes of road accidents, as well as determining actions to improve road safety at both local and global levels. The authors’ research demonstrated that integrating various strategies can contribute to reducing the number of fatal and serious accidents on roads. Measures to improve road safety should include reducing accident risks, preventing accidents, minimizing bodily injuries, and enhancing post-accident medical care.

Article [38] discusses the main factors contributing to road accidents, focusing on collisions involving young and elderly drivers. The study is based on the opinions of police officers, drivers, and official documents related to road accidents. The results revealed that the views of experts and drivers align with the typical factors recorded in official documents related to accidents. Additionally, potential shortcomings in accident registries were uncovered, suggesting the need to update report forms to encompass the full range of factors contributing to road accidents. The authors’ conclusions indicate the need to minimize delays in reporting accidents, suggesting the utilization of mobile reporting devices at the scene of incidents.

Article [39] presents methods for predicting road accidents within the Driver–Vehicle–Road–Environment (DVRE) system. By utilizing statistical forecasting methods, an assessment of the impact of various factors on accident rates was conducted, allowing for an evaluation of the effectiveness of the proposed measures aimed at improving road safety. The authors propose an integrated approach to effectively study accident concentration sites, incorporating accident forecasting methods.

Modeling the daily number of road accidents, as in article [40], is essential not only for insurance companies but also for institutions such as national road administrations or national insurance bureaus. According to the authors, accurate accident forecasts can bring benefits through the efficient planning of remedial actions, the optimization of reservation processes, better capital allocation, and the maintenance of roads. Therefore, it is crucial to develop a realistic model for predicting the daily number of road accidents. A key element of this model is the consideration of daily seasonality, characterized by a long period.

Article [41] analyzes the complexity of the causes of road accidents, noting that a single specific cause cannot unequivocally determine the entire event. Road accidents result from many interacting factors, with most classification systems focusing mainly on the errors and actions of participants. The authors’ analysis focuses on the most common factors contributing to road accidents in selected risk groups, such as young drivers, seniors, and risky drivers.

Article [42] examines the use of advanced techniques and algorithms for predicting road accidents, classifying data sources, and comparing different algorithms. The authors emphasize that combining various analytical techniques yields the best results. Future challenges include incorporating heterogeneous data sources, which may improve forecast accuracy.

Road traffic accidents are a global health issue, particularly severe in low- and middle-income countries, where approximately 1.19 million people die annually. Article [43] provides an overview of this phenomenon and important precautionary measures and prevention strategies, emphasizing the need for effective intervention to minimize the risk of road accidents and promote safe roads.

Article [44] analyzes the effectiveness of various machine learning models in predicting the severity of road accidents in New Zealand based on data from 2016–2020. The authors compare models such as Random Forest, Decision Jungle, Adaptive Boosting, XGBoost, Light Gradient Boosting Machine, and CatBoost. The results indicate that Random Forest achieves the highest accuracy, at 81.45%. Shapley analysis helps identify significant factors affecting the severity of injuries, such as road category and number of vehicles.

Article [45] conducts an analysis of the severity of road accidents in 30 cities in India, based on a two-stage MCDM model, which includes the Analytic Hierarchy Process (AHP) and multi-objective optimization based on the coefficient analysis (MULTIMOORA). Data on road accidents were provided by the Indian Ministry of Road Transport and Highways. This model relies on experts to determine the weights of different types of injuries and generates city rankings based on integrated Injury Severity Values (IIS). Additionally, the two-stage analysis of injury severity (PIIS) classifies cities into three stages based on IIS. The results indicate that Delhi, Chennai, Jaipur, and Bengaluru are cities with the highest IIS values, in the first phase of severity. This model has the potential to support decision-making regarding road safety and prioritize intervention programs.

Article [46] examines factors influencing fatal road accidents involving pedestrians and motor vehicles in the Silesian Voivodeship in the years 2016–2021. The model identified key features increasing the risk of pedestrian fatalities, including driving under the influence of alcohol and speed, especially involving heavy vehicles, at night, outside built-up areas, and in adverse weather conditions. These results may be useful in improving pedestrian safety.

Furthermore, it should be noted that lumbar spine injuries are well-researched in the literature, but there are few studies focusing on body dynamics in a reclined position. The diversity of seat belt reactions and altered body dynamics can negatively affect the effectiveness of airbags, especially when dealing with submersion, which increases the risk of head injuries due to the greater distance from the airbag [47,48,49].

Modern road safety and the effectiveness of protective equipment in vehicles are key issues. A review of the literature studies indicates that the identification and elimination of the main factors leading to road accidents is crucial for the development of effective prevention strategies. Crash tests are an indispensable part of assessing vehicle safety and communicating needed design improvements. Technologies such as emergency braking systems and lane assistants significantly reduce the number of accidents. Legislation plays an important role in improving road safety, and its continuous development is essential. Various articles provide detailed conclusions, such as the need to integrate multiple strategies to reduce fatalities and serious injuries. Accurate accident reporting and the use of mobile reporting devices can improve data quality. Traffic accident prediction models help you plan and optimize your safety efforts. Machine learning models like Random Forest are effective at predicting the severity of accidents. An analysis of accident data from cities can support decision-making and the prioritization of safety interventions. Understanding the factors contributing to fatal accidents involving pedestrians can lead to improved safety for the most vulnerable road users.

A particularly important element is to determine the impact of the seat position and headrest backrest on the safety of the driver and passengers. In addition, an important parameter is to determine the impact of these parameters on the behaviour of the human body during crash tests at low speed. The development of road infrastructure, including the expansion of motorways or expressways, reduces the likelihood of head-on and side accidents, because vehicles move only in one direction. Also in cities, where an extensive road network allows vehicles to move in several lanes at the same time in the same direction, reduces the risk of a head-on accident. The development of infrastructure is conducive to the occurrence of rear accidents, especially when rear-ending the vehicle at an intersection without braking. Most collisions in traffic jams occur at low speed. Therefore, it is crucial to learn about the behaviour of the human body during collisions at low speed. This chapter highlights the importance of a multidisciplinary approach to improving road safety and the need for continuous research and innovation in this area.

## 3. Experimental Studies

In order to study the behaviour of the human body during a collision at a low speed of 20 km/h, the authors had to carry out a series of actions. First, a test stand was designed, with a platform on which the car seat was placed. The next step was to build an anthropometric dummy that could be used for rear, side and frontal crash tests—at low crash speeds. The next step was the crash tests of volunteers on the test bench. In the next step, the built dummy, together with its simulation counterpart made in the MSC ADAMS program, was compared with volunteers and the HYBRID III dummy during crash tests at low speed. Only the KPSIT C50 dummy and the KPSIT C5 dummy, which were compared and validated with the validated dummy, were used for crash tests at low speed. In this article, the authors investigated how the angle of inclination of the backrest of a passenger car seat affects the displacement of the head during a collision. By reviewing the scientific literature, it can be concluded that the inclination of the backrest affects the displacement of the dummy’s body during the crash test. However, it should be noted that these types of crash tests were performed at higher crash speeds—most often from 30 km/h to 64 km/h. It should be noted that the dummies available on the market are not dedicated to a low-speed collision of up to 25 km/h. Manufacturers of anthropometric dummies, along with the purpose of the dummy, determine at what crash speeds crash tests should be performed. Therefore, for the authors, the key element was to make a dummy for low-speed collisions. By performing a series of crash tests, the authors wanted to check whether the change in the angle of the car seat backrest is also an important parameter during a low-speed collision.

- Experimental Design

**Figure 1:**
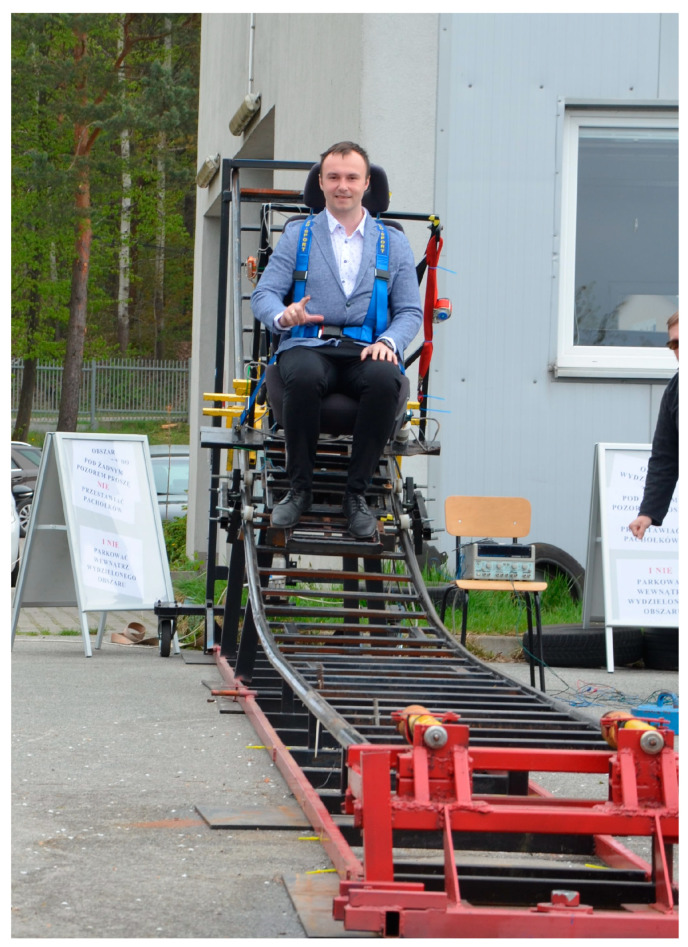
Low-speed crash test simulation bench.

Crash tests were carried out on a test stand belonging to the Laboratory of Vehicles and Tractors, Department of Motor Vehicles and Transport. The KPSIT C50 dummy was used in the study, designed to record body movements during low-speed collisions. The study was conducted in high sunlight. A high-speed Digital Phantom V310 camera was used to record the fast-changing phenomenon. The crash test simulation rig is shown in Figure 1. The stand allows you to record collisions from a speed of 5 km/h to 25 km/h. The tests carried out for the purposes of this study were carried out at a collision speed of 20 km/h.

b. Implementation of Experimental Research

**Figure 2:**
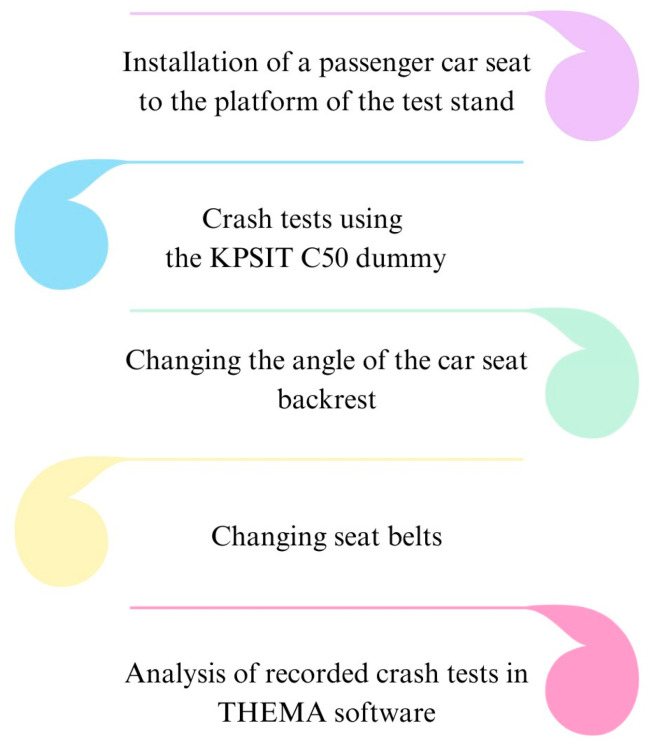
Simplified test procedure.

Firstly, during the research, a car seat was selected and installed in the laboratory setup. The chosen car seat was from a Hyundai I30 vehicle and was secured to the platform using steel screws. Subsequently, a control measurement of the recline angle of the car seat was performed, with a value of 100° established. Then, a KPSIT C50 dummy was placed on the seat. The dummy represents the 50th percentile of the male population. In the next step, a series of 5 crash tests were conducted at a speed of 20 km/h. The displacement results of the dummy’s body parts were averaged based on this series of five measurements for analysis purposes. Following this, the recline angle of the car seat was changed by 5°. Crash tests were conducted in the range of recline angles from 100° to 125°. After completing the crash test series, the standard three-point seatbelt was replaced with a four-point seatbelt. The entire crash test procedure was repeated after the belt replacement. All conducted crash tests were processed and analyzed using THEMA software. A simplified schematic of the research procedure is presented in Figure 2.

c. Data Analysis

**Figure 3:**
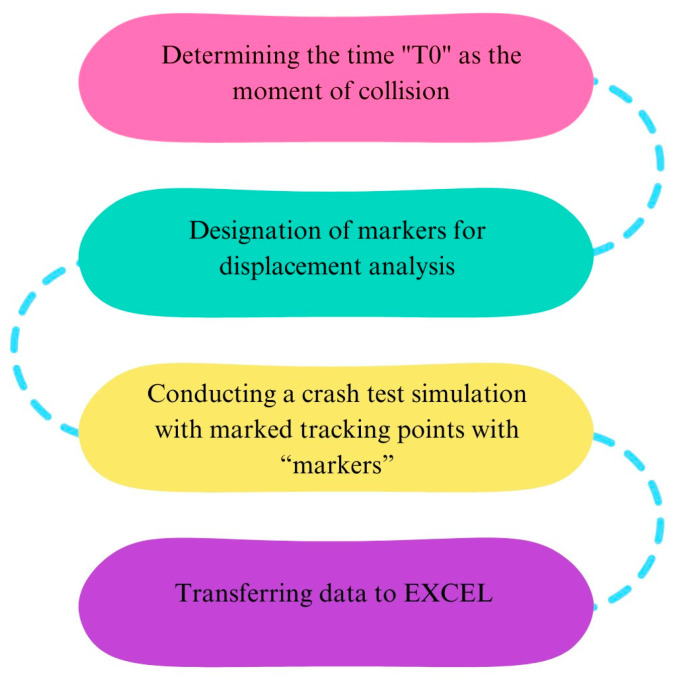
Workflow for recorded data.

As part of the research procedure, crash tests were recorded with an accuracy of 2500 frames per second. All recorded videos have been processed in THEMA. The first step in the analysis was to determine the moment “T0” as the time of the collision. Then, the markers in the program were marked. During the crash tests, markers were placed on the head, neck, shoulders, knees of the dummy and on the elements of the stand, such as the car seat and headrest. The next step was to simulate a crash test—in this step, the program tracked the trajectories of the individual elements of the dummy and the stations on which the markers were located. After the analysis in THEMA, the data was converted to EXCEL in order to make displacement diagrams. A diagram of how to handle the recorded data is shown in Figure 3.

d. Object of Experimental Research

**Figure 4:**
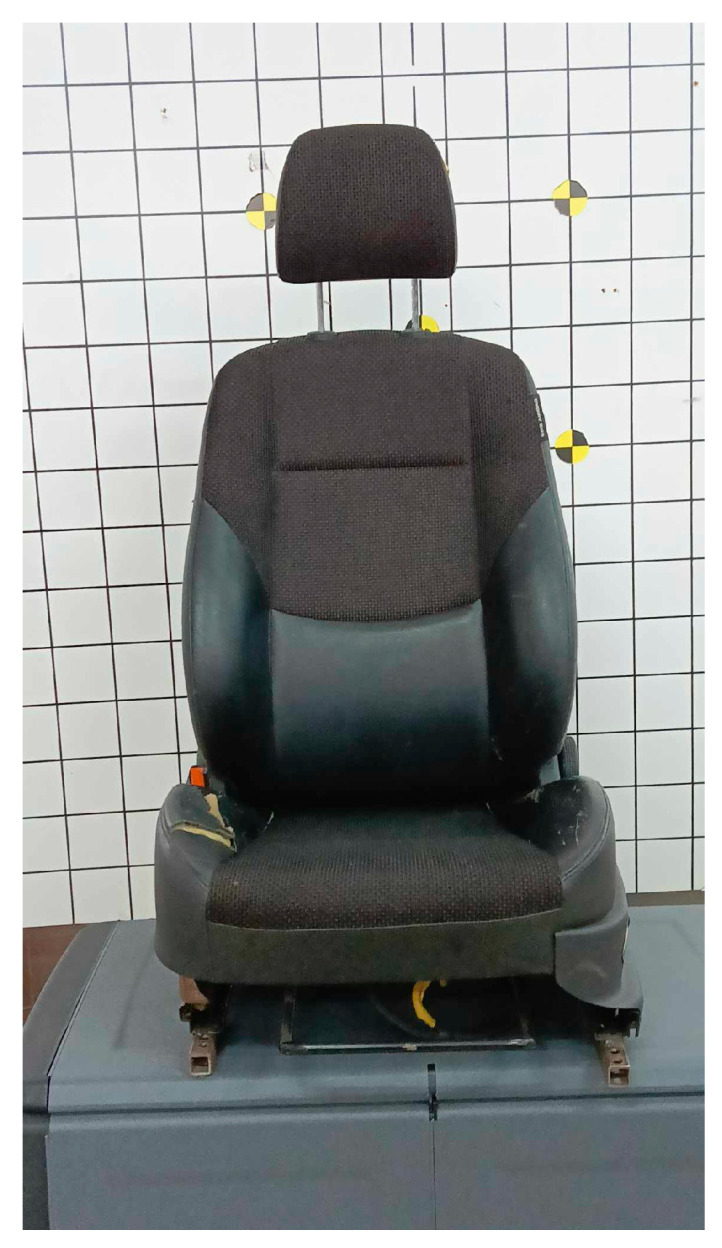
Passenger vehicle seat.

During the research, a car seat from the Hyundai I30 car was used. The armchair is shown in Figure 4. The main objective of the research is to learn about the trajectory of the dummy’s head at the moment of a collision of 20 km/h when changing the angle of the car seat backrest and changing the seat belt. The trajectory of the head will help us determine the risk of potential injury to a human being when the car seat is incorrectly positioned at low crash speed.

**Figure 5:**
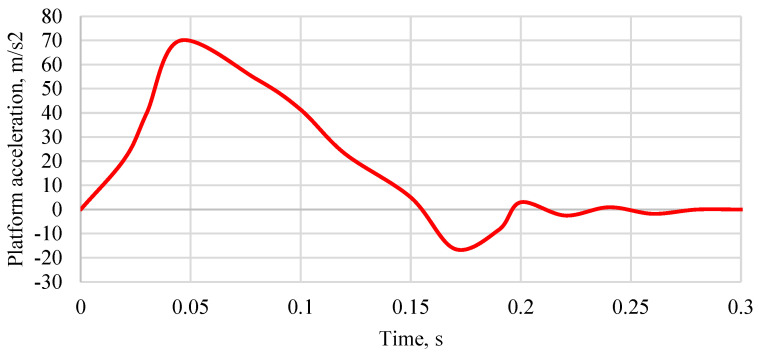
The course of acceleration of the platform with the vehicle seat during experimental tests.

**Figure 6:**
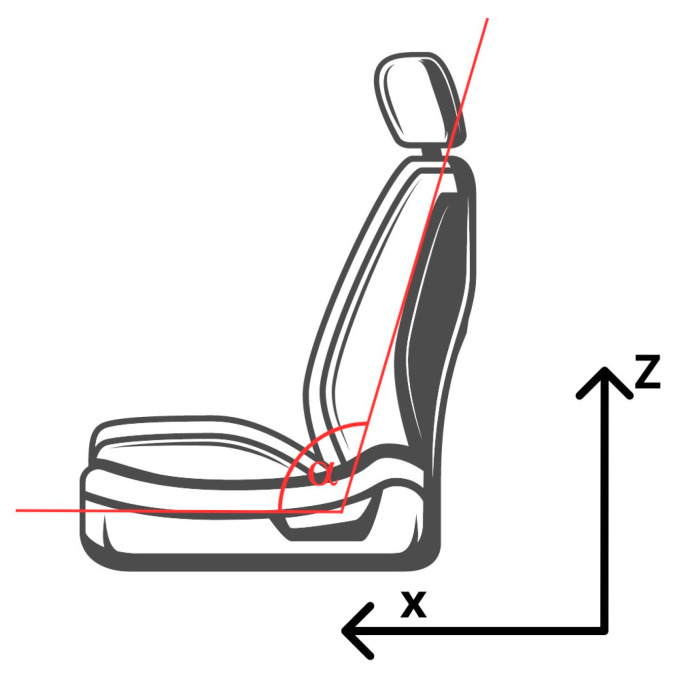
Adopted measurement coordinate system.

For the sake of safety and reproducibility of the results, each time after the crash test, the repeatability of the acceleration impulse of the platform with the chair and the dummy was analyzed. The benchmark was the average deceleration of the bogie from five crash tests, which was 69.68 m/s^2^. The acceleration of the platform with the vehicle seat during the experimental tests is shown in Figure 5. Platform acceleration pulse values that deviated from the assumed nominal value by more than 10% were rejected. The roller bearings of the platform were then cleaned or replaced with new ones and the test was repeated. The adopted coordinate system is shown in Figure 6. Due to the use of a single high-speed camera, displacements in the direction of the *Y*-axis were not analyzed.

## 4. Crash Test

**Figure 7:**
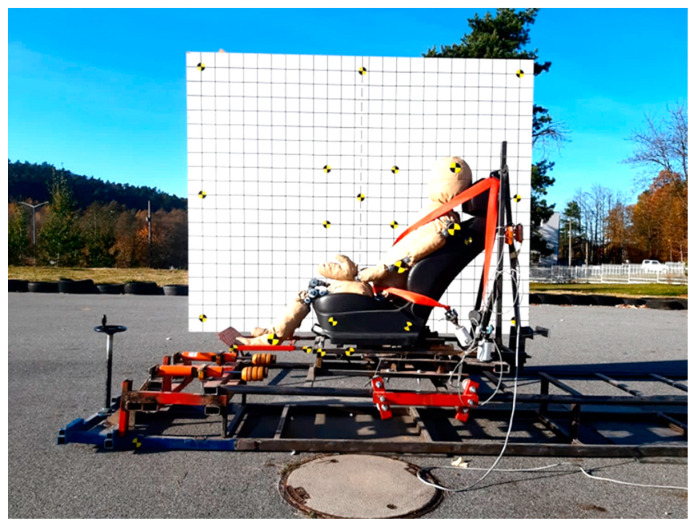
Dummy during a crash test of 20 km/h with a backrest angle of 125° and three-point belts [52].

The KPSIT C50 dummy was used in the study, which in the publications [50,51] was compared with the KPSIT C50 simulation dummy and the Hybrid III physical dummy during the 20 km/h crash test. Figure 7 shows the dummy during the crash test with a backrest angle of 125° and three-point belts.

**Figure 8:**
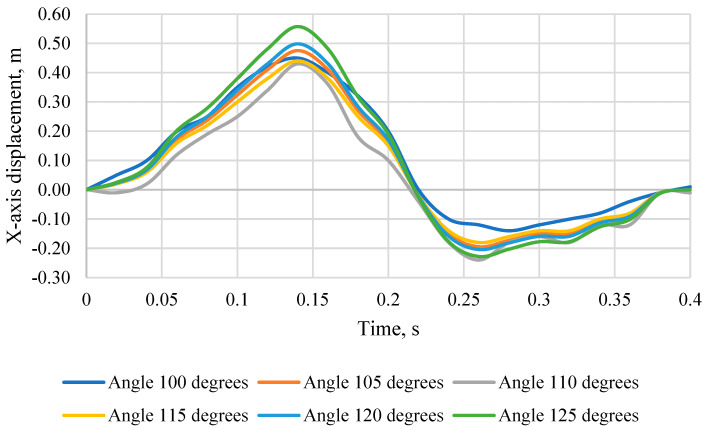
Displacement of the head of the KPSIT C50 dummy in the direction of the *X*-axis—during the crash test with three-point belts and the range of the seat backrest angle from 100 to 125 degrees.

Figure 8 shows the head displacement of the KPSIT C50 dummy towards the *X*-axis during a 20 km/h crash test, changing the seat backrest angle from 100 degrees to 125 degrees and using three-point belts. When comparing the displacement of the head relative to the *X*-axis for the different angles of the chair, you can see that it varies depending on the angle at which the backrest is located. Assuming the optimal angle of inclination of the chair equal to 110 degrees, it can be seen that for the angle of 100°, the maximum displacement is 0.45 m and it occurs at the time of 0.14 s, for the angle of 105° it is 0.44 m and occurs at the time of 0.14 s, for the angle of 110° it is 0.43 m and occurs at the time of 0.14 s, for the angle of 115° it is 0.44 m and occurs at the time of 0.14 s, for an angle of 120° it is 0.50 m and occurs at a time of 0.14 s, while for an angle of 125° it is 0.56 m and occurs at a time of 0.14 s. When analyzing the maximum displacement of the head backwards, which is expressed as negative values, it can be seen that it also varies depending on the angle of the chair. For the angle of 100° the maximum displacement towards the back is −0.14 m and it occurs at the time of 0.26 s, for the angle of 105° it is −0.18 m and occurs at the time of 0.26 s, for the angle of 110° it is −0.24 m and occurs at the time of 0.26 s, for the angle of 115° it is −0.18 m and occurs at the time of 0.26 s, for the angle of 120° it is −0.20 m and occurs at the time of 0.26 s, while for an angle of 125° it is −0.23 m and occurs at a time of 0.26 s.

**Figure 9:**
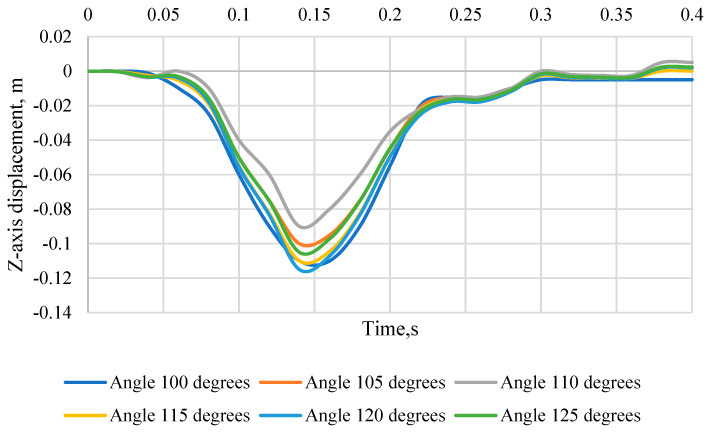
Displacement of the head of the KPSIT C50 manikin in the direction of the *Z*-axis—during the crash test with three-point belts and the range of the seat backrest angle from 100 to 125 degrees.

Figure 9 shows the head movement of the KPSIT C50 *Z*-axis during a 20 km/h crash test, changing the seat backrest angle from 100 degrees to 125 degrees and using three-point belts. Negative values indicate downward displacement, while positive values indicate upward displacement. For each angle of inclination of the chair, you can see the change in displacement over time. It is worth noting that the displacement is greatest for the 110° angle and the 115° angle, and the smallest for the 125° angle. The time it takes for the maximum displacement to occur varies depending on the angle of the chair. For example, for an angle of 110°, the maximum displacement occurs at 0.1 s, while for an angle of 120° it occurs at 0.1 s. At the same time, for all tested chair inclination angles, the head of the KPSIT C50 dummy moves downwards. The exact displacement values for each angle are as follows: for the angle 100° −0.11 m, for the angle 105° −0.1 m, for the angle 110° −0.09 m (the smallest displacement), for the angle 115° −0.11 m, for the angle 120° −0.115 m, and for the angle 125° −0.105 m (the largest displacement).

**Figure 10:**
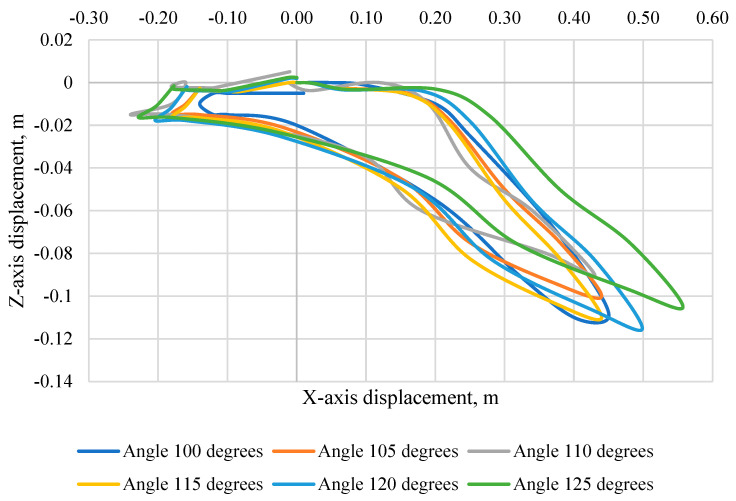
Head trajectory of the KPSIT C50 dummy—during the crash test with three-point belts and the range of the seat backrest angle from 100 to 125 degrees.

Figure 10 shows the trajectory of the KPSIT dummy’s head during a frontal crash test at a speed of 20 km/h and the change in the angle of the seat backrest from 100 degrees to 125 degrees using three-point belts. The largest displacement towards the *Z*-axis was recorded in a collision with a backrest angle of 120 degrees (−0.115 m). In addition, the largest displacement in the direction of the *X*-axis was recorded at an angle of 125 degrees (0.56 m) and the smallest at an angle of 110 degrees (0.43 m). We can also notice that with a backrest angle of 115 degrees, 120 degrees and 125 degrees, there is rotation in the final phase of the head movement, which can be the cause of damage.

**Figure 11:**
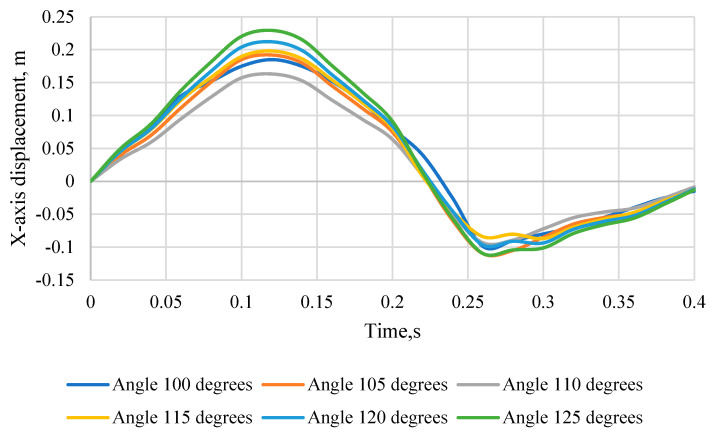
Displacement of the head of the KPSIT C50 dummy in the direction of the *X*-axis—during the crash test with four-point belts and the range of the seat backrest angle from 100 to 125 degrees.

Figure 11 shows the head movement of the KPSIT C50 dummy in the *X*-axis during a 20 km/h crash test, changing the seat backrest angle from 100 degrees to 125 degrees and using four-point belts. When comparing the displacement of the head in the direction of the *X*-axis for different angles of the chair, it is possible to notice their differentiation depending on the position of the backrest. The displacement values change with the angle of the chair. For an angle of 100° the maximum displacement is 0.185 m and it occurs at a time of 0.14 s, for an angle of 105° it is 0.19 m (time: 0.14 s), for an angle of 110° it is 0.16 m (time: 0.14 s), for an angle of 115° it is 0.2 m (time: 0.14 s), for an angle of 120° it is 0.21 m (time: 0.14 s), and for an angle of 125° it is 0.23 m (time: 0.14 s). When analysing the displacement of the head in the direction of the *X*-axis, it is worth noting that negative values indicate the maximum displacement of the head backwards. For an angle of 100° at a time of 0.24 s, the displacement is −0.026 m, for an angle of 105° at the same moment it is −0.06 m, for an angle of 110° it is −0.05 m, for an angle of 115° it is −0.05 m, for an angle of 120° it is −0.05 m, and for an angle of 125° it is −0.06 m. These negative values suggest that the dummy’s head has moved backwards, which may be relevant to assessing potential injuries in the event of collisions. Therefore, both positive and negative displacement values should be taken into account when analysing crash test data.

**Figure 12:**
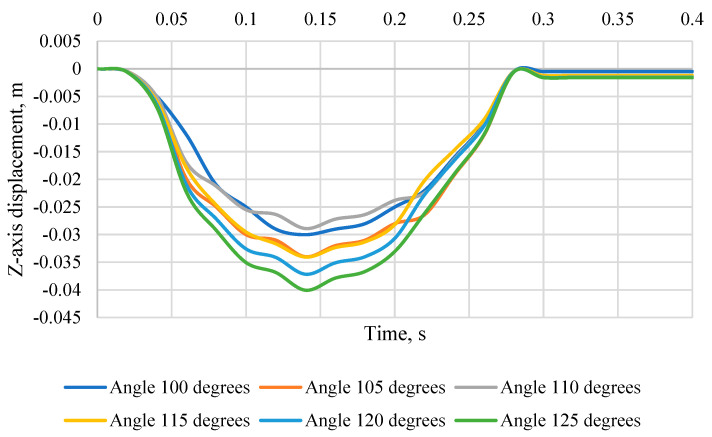
Displacement of the head of the KPSIT C50 dummy in the direction of the *Z*-axis—during the crash test with four-point belts and the range of the seat backrest angle from 100 to 125 degrees.

Figure 12 shows the head movement of the KPSIT C50 *Z*-axis during a 20 km/h crash test and the seat backrest angle changed from 100 degrees to 125 degrees while using four-point belts. When analysing the movement of the dummy’s head towards the *Z*-axis, i.e., upwards, it can be seen that the data present differences depending on the angle of the chair. At the time of 0.14 s, when the head reaches its maximum forward displacement during the crash test, the displacement of the dummy head relative to the *Z*-axis for different angles is: for 100°: −0.03 m, for 105°: −0.034 m, for 110°: −0.0289 m, for 115°: −0.034 m, for 120°: −0.0378 m, and for 125°: −0.040 m. These negative values indicate that the dummy’s head is moved upwards at the moment of maximum forward movement during the collision. It is worth noting that the greater the angle of inclination of the chair, the greater the displacement in the direction of the *Z*-axis.

**Figure 13:**
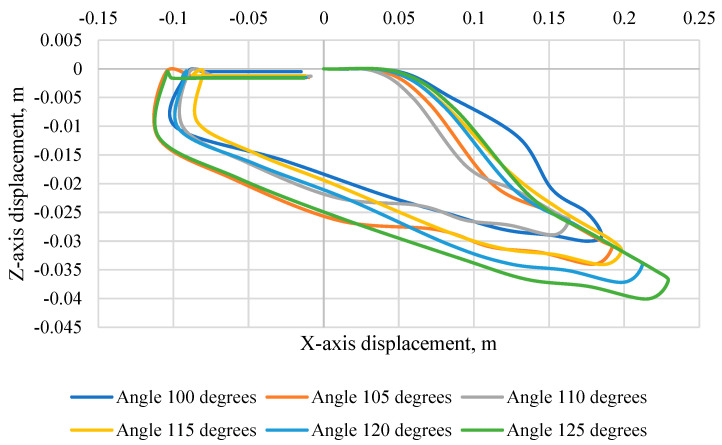
KPSIT dummy’s head trajectory—during the crash test with four-point belts and the seat backrest angle range from 100 to 125 degrees.

Figure 13 shows the trajectory of the KPSIT dummy’s head during a frontal crash test at a speed of 20 km/h and the change in the angle of the seat backrest from 100 degrees to 125 degrees using four-point belts. The largest displacement in the *Z*-axis direction was recorded during a collision with a backrest angle of 125 degrees (−0.04 m). In addition, the largest displacement in the direction of the *X*-axis was recorded at an angle of 125 degrees (0.23 m) and the smallest at an angle of 110 degrees (0.15 m).

## 5. Discussion

An article [53] analyses the head movement of adult passengers in the front seat during sudden vehicle manoeuvres. The authors collected data from 87 men and women of different physical characteristics and ages on a closed test track. Participants were instructed that the study was about vehicle dynamics. The results showed significant head tilt when braking and changing lanes. The study was the first quantitative analysis of passenger dynamics to take into account sample diversity, which allowed the study to investigate the effect of age and physical characteristics on head movement.

In this publication, we have confirmed that head displacement during a 20 km/h collision is related to the position of the car seat and the use of the seat belt.

The study carried out by the authors of the article [54] focuses on the consequences of unusual positions of passengers in the front seats during car accidents, which is important in the context of the potential introduction of autonomous vehicles. By modeling the front seat of the SUV, accidents were simulated to represent situations at intersections. Kinematics and body loads in different passenger positions were analysed. The results showed that changes in lower limb posture had the greatest impact on the body’s response during accidents. Crossing the legs during frontal collisions caused the greatest deflection and rotation of the pelvis, which significantly affected the behaviour of the whole body. In side impacts, the tilt of the torso had a key effect on the kinematics of the head and torso, altering the interaction with the interior of the vehicle.

In this article, only the head displacement of the KPSIT C50 dummy was analyzed. Based on the results, it should be noted that the use of four-point belts reduces the displacement of the dummy’s head by more than 50% compared to the displacement of the head during a collision with the standard three-point belts.

The paper [55] analyzed passenger reactions during simulated collisions of future autonomous vehicles, focusing on seat configuration. In the first part of the simulation, a head-on collision was carried out, where the passengers in the front seats had their seats turned upside down and one of them was not wearing a seatbelt. The second part of the study investigated occupant safety in different seating configurations, using a single-seat model with a three-point seat belt. The results showed that passengers in the front seats, rear-facing, were held down by the backrest of the seat, restricting the forward movement of their torso. In contrast, the passengers in the rear seats, wearing seatbelts, had a greater displacement of their body as a result of the accident. In the second part of the study, it was found that the safety of passengers depends on the direction of the seat. The backrest of the seat generated a contact force with the passenger, limiting his displacement. The relaxed position allowed for more lean compared to the driving position, especially when the passenger was rear-facing at a lower impact speed.

This research confirms that the most relaxed position, i.e., the one in which the chair is most reclined, is the least safe. On the basis of his own research, the author noticed that during crash tests, when the chair was reclined from 115 to 125°, there was a rotation of the head in the final stage of the collision, when the head of the dummy moved as far back as possible and the head moved forward again. The rotation occurred before the stabilization phase, which occurs after 0.26 s of impact.

In the article [56], in six tests at a speed of 48 km/h and a lean angle of 195 degrees, the various front seats on which the ATD dummies were placed were tested. Tests showed that the seats shifted and twisted during impact. The movement of the torso relative to the backrest of the seat was restricted, which resulted in the possibility of protruding the head and neck over the backrest of the seat. The angle of inclination of the seat backrest was greatest for one of the seats, and the backward deformation time was longer for the seats, which provided greater deformation. The biomechanical responses were in most cases below the limits of the reference values for injury. However, higher tensile moments in the lower neck were observed in some chairs, indicating a potential risk of injury. The conclusion of the authors’ study is that chairs with higher deformation provided longer energy absorption time and lower overall biomechanical responses. However, the lack of head support during strong rear-end impacts can lead to a strong lower neck response, highlighting the importance of energy management in seat design.

In the study, the author points out that during a frontal collision at a low speed not exceeding 6 m/s, no serious injuries occur. However, incorrect positioning of the seat and moving the seat too close to the steering wheel and cockpit of the vehicle can cause us to hit our head on these elements. Studies have shown that when the seat is correctly positioned (110°), the head moves by an average of 0.45 m in a 20 km/h collision. Therefore, there is a high risk of hitting your head against the cockpit of the vehicle or the steering wheel.

In article [57], a series of crash tests of the seat belt buckle were performed. In order to investigate the kinematics of the driver and the interaction of the passenger with the seat belts, two controlled tests were carried out on a sled in a frontal collision with a vehicle equipped with an airbag. The tests included situations where the seat belts were fully or partially fastened. The results of the tests showed that when the seat belts were partially immobilized after the buckle was released, the tape became entangled with the left arm of the anthropometric dummy (ATD), which generated high contact forces on the shoulder, which could lead to significant surface injuries. The authors’ research suggests the importance of properly fastening a seatbelt while traveling, especially when combined with the presence of airbags, to reduce the risk of injury in the event of an accident. In addition, the study carried out in the article [58] aimed to investigate the effect of lumbar and pelvic/spine angles on the effectiveness of seat belts in reducing injuries from road accidents. Despite analyses using models of the human body, no relationship between these angles and the effectiveness of seat belts has been demonstrated so far. The study measured lumbar and pelvic/spine angles in 75 people and then used them to create three different models for the total human model in terms of safety. The simulation results showed that as lumbar lordosis increases and pelvic angle decreases, the seat belt is more likely to catch on the hip bone, which can make it more difficult to cause injury. This finding could have important implications for rethinking the optimal positioning of the seat and seat belt to increase the effectiveness of protection in the event of road accidents.

In the present study, a smaller displacement of the head of the KPSIT dummy was demonstrated during a collision with the use of four-point belts. Four-point belts do not have a seat belt pretensioner, so they rigidly maintain the positions of the dummy’s body. In the case of low-speed collisions, when the greatest threat is the movement of the human body, the use of four-point belts seems to be a better solution.

Studies have shown that the correct angle of the seat backrest between 110° and 115° and the use of four-point belts reduce head displacement and better protect occupants at low crash speeds. The proper fastening of seat belts and the proper configuration of seats are key to reducing the risk of injury.

## 6. Conclusions

The movement of the head towards the *X*-axis in a frontal collision, with the three-point seat belt used, varies depending on the angle of the seat. The displacement values are shown in meters. An analysis of the data shows that for the seat angles of 100° to 125°, the displacement of the head changes during the collision. In general, head displacement usually increases as the angle of the chair increases. For most seat angles (e.g., 105°, 110°, 115°), head displacement increases with the duration of the impact. However, there are some cases, for example for a 100° angle, where the displacement of the head seems to decrease slightly at some point in the collision and then increases again. These differences may be related to the different forces acting on the body depending on the angle of the seat and the changing conditions during the collision. Analysing this data can be useful for designing more effective vehicle safety systems and for understanding the impact of different seat settings on occupant behaviour during road accidents.

Comparing the displacements of the head in the direction of the *X*-axis for different angles of the chair with respect to the angle of 110 degrees, which we take as a reference point (0), the following differences can be noticed: For an angle of 100 degrees, the displacement is 0.02 m, which is a difference of 0.02 m from an angle of 110 degrees. For an angle of 105 degrees, the displacement is 0.06 m, which is a difference of 0.06 m with respect to an angle of 110 degrees. For an angle of 115 degrees, the displacement is 0.16 m, which is a difference of 0.16 m with respect to an angle of 110 degrees. For an angle of 120 degrees, the displacement is 0.22 m, which is a difference of 0.22 m with respect to an angle of 110 degrees. For an angle of 125 degrees, the displacement is 0.25 m, which is a difference of 0.25 m with respect to an angle of 110 degrees. These differences illustrate how the displacement of the head changes depending on the angle of the chair compared to the value for the angle of 110 degrees.

Studies have shown that both at speeds of 20 km/h and higher speeds, the position of the passenger car seat affects the trajectories of the dummy’s movement. Therefore, the recommended angle of 110° between the seat and the backrest is a safe range for the car seat. In further research, the author will conduct low-speed crash tests along with the change in the angle of impact and check the effect of the four-point and five-point belts used on the trajectories of the dummy’s head during side and rear collisions.

The research problem posed in this work has been solved. The angle of the seat backrest has a large impact on the displacement of the dummy’s head during a collision at a speed of 20 km/h. Statements from the literature regarding crash tests at higher speeds above 30 km/h have been confirmed; with the backrest angle above 115° there is rotation of the head, which can cause serious injuries in the upper cervical region. In his subsequent works, the author will expand the upper cervical spine of the dummy with an additional joint, along with force and acceleration sensors, which will allow the author to specify how the angle of the seat backrest can affect injuries of the upper cervical part during a low-speed collision. In addition, the author will further study how the angle of the seat backrest affects the displacement of the individual parts of the dummy’s body, taking into account low-speed, side and rear crash tests.

## Data Availability

Data from conducted surveys and observational studies may be made available after sending an inquiry to the authors.
